# Liquid State Machine on SpiNNaker for Spatio-Temporal Classification Tasks

**DOI:** 10.3389/fnins.2022.819063

**Published:** 2022-03-14

**Authors:** Alberto Patiño-Saucedo, Horacio Rostro-González, Teresa Serrano-Gotarredona, Bernabé Linares-Barranco

**Affiliations:** ^1^Department of Electronics Engineering, University of Guanajuato, Salamanca, Mexico; ^2^Instituto de Microelectrónica de Sevilla (IMSE-CNM), Consejo Superior de Investigaciones Científicas (CSIC) and Univ. de Sevilla, Seville, Spain; ^3^Université de Lorraine, BISCUIT - Laboratoire Lorraine de Recherche en Informatique et ses Applications (LORIA), UMR 7503, Nancy, France

**Keywords:** Liquid State Machine, N-MNIST, neuromorphic hardware, spiking neural network, SpiNNaker

## Abstract

Liquid State Machines (LSMs) are computing reservoirs composed of recurrently connected Spiking Neural Networks which have attracted research interest for their modeling capacity of biological structures and as promising pattern recognition tools suitable for their implementation in neuromorphic processors, benefited from the modest use of computing resources in their training process. However, it has been difficult to optimize LSMs for solving complex tasks such as event-based computer vision and few implementations in large-scale neuromorphic processors have been attempted. In this work, we show that offline-trained LSMs implemented in the SpiNNaker neuromorphic processor are able to classify visual events, achieving state-of-the-art performance in the event-based N-MNIST dataset. The training of the readout layer is performed using a recent adaptation of back-propagation-through-time (BPTT) for SNNs, while the internal weights of the reservoir are kept static. Results show that mapping our LSM from a Deep Learning framework to SpiNNaker does not affect the performance of the classification task. Additionally, we show that weight quantization, which substantially reduces the memory footprint of the LSM, has a small impact on its performance.

## 1. Introduction

Neuromorphic computing research aims to enable the design of highly efficient devices capable of processing multi-scale and event-driven dynamic data, inspired by the ability of nervous systems in animals to coordinate actions with a vast stream of sensory information. At its core is the study of Spiking Neural Networks (SNNs), models that describe the dynamics and interactions of biological neurons, characterized by a spike time-encoding mechanism, event-based communication, and high parallelism. SNNs are being investigated in pattern recognition applications, and recent results show that they are able to match the performance of Deep Neural Networks in several computer vision and signal processing tasks (Tavanaei et al., 2019). Concurrently, efforts to deploy their time-encoding feature in hardware have resulted in the development of large-scale neuromorphic chips such as TrueNorth (DeBole et al., 2019), Loihi (Davies et al., 2018), and SpiNNaker (Furber et al., 2014).

Several works show a reduction in the power consumption of neuromorphic computing systems as opposed to conventional systems (CPU, GPU) for specific pattern recognition tasks (Diehl et al., 2016a; Amir et al., 2017; Liu et al., 2018) and optimization problems (Davies et al., 2021). This advantage would provide an edge of SNNs and neuromorphic computing in applications that require low power or high autonomy sensing and processing of data such as robotics, autonomous driving, and edge computing.

In the spectrum of Deep Neural Network architectures, Recurrent Neural Networks (RNNs) are among the most used for sequential and temporal processing, showcasing a high performance in machine translation (Sutskever et al., 2014), image captioning (Karpathy and Fei-Fei, 2015), speech recognition (Graves and Schmidhuber, 2005), time series prediction (Sagheer and Kotb, 2019), etc. (Lipton et al., 2015). Furthermore, recurrent connectivity is prevalent in biological brain modules (Lukoševičius and Jaeger, 2009). This makes the design of Recurrent Spiking Neural Networks (RSNNs) and their implementation on neuromorphic hardware an interesting area to explore for the development of more efficient machine learning solutions.

The Liquid State Machine (LSM) is one type of recurrently connected network of spiking neurons. Proposed by Maass et al. (2002), LSMs are randomly generated recurrent spiking neural networks whose internal connectivity parameters remain static during the training process, acting as a *reservoir*. This reservoir is excited by input signals and its state, a non-linear transformation of the input's history, is connected to a linear readout unit. The state of the reservoir can be seen as a mapping of the input data into a higher dimension where the prediction or classification task is easier to solve, similar to the kernel methods like Support Vector Machines. The main hypothesis regarding this kind of network is that, if set properly, they are able to represent spatiotemporal inputs in a higher dimensional space where non-linear combinations of frequencies resonate, providing useful information that makes the characterization of the input simpler to infer, while requiring a significantly less amount of computational resources compared to an RSNN trained in a standard way (Cramer et al., 2020), as the connectivity weights among layers of the network are not trained, except for the output or classification layer.

While RSNNs seem an obvious design choice for neuromorphic computing platforms, very few works have attempted to implement large-scale Spiking RNNs in neurosynaptic processors. Diehl et al. (2016b) implemented an Elman RNN for question classification in the IBM's TrueNorth using a “train-and-constrain” methodology including a 16-level weight quantization. The inputs were converted to spikes through a simple rate encoding. Shrestha et al. (2018) used a similar approach, that involves approximation techniques such as activation discretization, weight quantization, scaling, and rounding, to implement an LSTM for sequence classification tasks.

In this article, we propose a method to train a Spiking RNN in a deep learning framework (Paszke et al., 2019), and to implement the trained model in a neuromorphic platform (Furber et al., 2014) for event classification. The model of choice is that of Liquid State Machines (LSMs). We use the Neuromorphic MNIST (N-MNIST) (Orchard et al., 2015) dataset to train and validate the results. The choice of an event-driven dataset instead of sequential datasets eliminates the need for spike conversion in our proposed method.

Related work includes that by Tian et al. (2021), who proposed a method to train an LSM using a neural architecture search which achieved a 92.5% accuracy for the N-MNIST without a hardware implementation, and (Yang et al., 2020), who trained an LSM for the N-MNIST dataset with 93.1% accuracy and deployed it in a custom 32 nm ASIC. The best-reported accuracy for the N-MNIST dataset trained with a Deep SNN corresponds to the work by Samadzadeh et al. (2020), who achieved 99.6% using a Spiking CNN with Residual blocks and spatio-temporal backpropagation. For this and the aforementioned related works, the accuracy is calculated as the percentage of correctly classified inputs on a test set of 10,000 samples, which are not used in the training process.

The main contributions of this work are:

- The design and implementation of an LSM for the SpiNNaker, a large-scale neuromorphic processor, which is widely used in neuromorphic computing research.
- State of the art results by an LSM for the N-MNIST dataset.
- Analysis of the impact of size and weight precision in the performance of the LSM in the SpiNNaker platform.

Results of this work show a 94.43% accuracy for the best LSM, which outperforms the state-of-the-art.

## 2. Materials and Methods

We propose an offline learning approach for implementing a functional Liquid State Machine on SpiNNaker, consisting of a two-stage pipeline. In the first stage, the LSM architecture is implemented in PyTorch (Paszke et al., 2019), which is used to train the hidden-to-readout weights, aided by the computing power of GPUs. In the second stage, the trained LSM architecture and parameters are mapped onto SpiNNaker, and comparisons are made with the PyTorch implementation for a classification task in an event-based dataset. In the subsequent sections, we will describe the neuron and network models of the proposed LSM, the training procedure, and the SpiNNaker implementation.

### 2.1. Neuron Model

The spiking neuron model used in this work is the Leaky Integrate-and-Fire (LIF) (Gerstner and Kistler, 2002), suitable for very efficient hardware implementations. The dynamics of the membrane potential *u*(*t*) of a single neuron is given by:


(1) $$\begin{array}{l} \frac{du\left(t\right)}{dt}=\frac{u_{rest}-u\left(t\right)}{\tau_{m}}+\frac{I\left(t\right)}{c_{m}} \end{array}$$

where *u*_*rest*_ is the resting potential, τ_*m*_ is the membrane's time constant, *c*_*m*_ is the membrane capacitance, and *I*(*t*) is the neuron's input or stimulus.

A network of spiking neurons is formed by at least a presynaptic and a postsynaptic neuron with membrane potentials *u*_*pre*_(*t*) and *u*_*pos*_(*t*), respectively. When the presynaptic neuron's membrane potential reaches a threshold *u*_*th*_, the neuron fires, its membrane potential is reset to *u*_*reset*_ and the spike stimulates the postsynaptic neuron through a current *I*(*t*), after a short synaptic delay. To further simplify the dynamics, we make *u*_*reset*_ and *u*_*rest*_ both equal to zero in our simulations.

The behavior of the postsynaptic membrane potential in discrete time, *u*_*pos*_[*k*] corresponds to an exponentially decaying function toward *u*_*rest*_, with a time constant τ_*m*_, perturbed by the presence of a presynaptic input *I*[*k*]. Considering a time resolution Δ*t* and the effect of membrane reset due to firing, the discrete update equation of the membrane potential in this model is:


(2) $$\begin{array}{l} u_{pos}[k]=\{\begin{array}{ll} u_{reset} & u_{pos}[k-1]\geq u_{th}\\ u_{pos}[k-1]e^{-\frac{\Delta t}{\tau_{m}}}+\frac{I[k-1]\Delta t}{c_{m}} & \text{otherwise} \end{array} \end{array}$$

### 2.2. Recurrent Network Model

**Figure 1:**
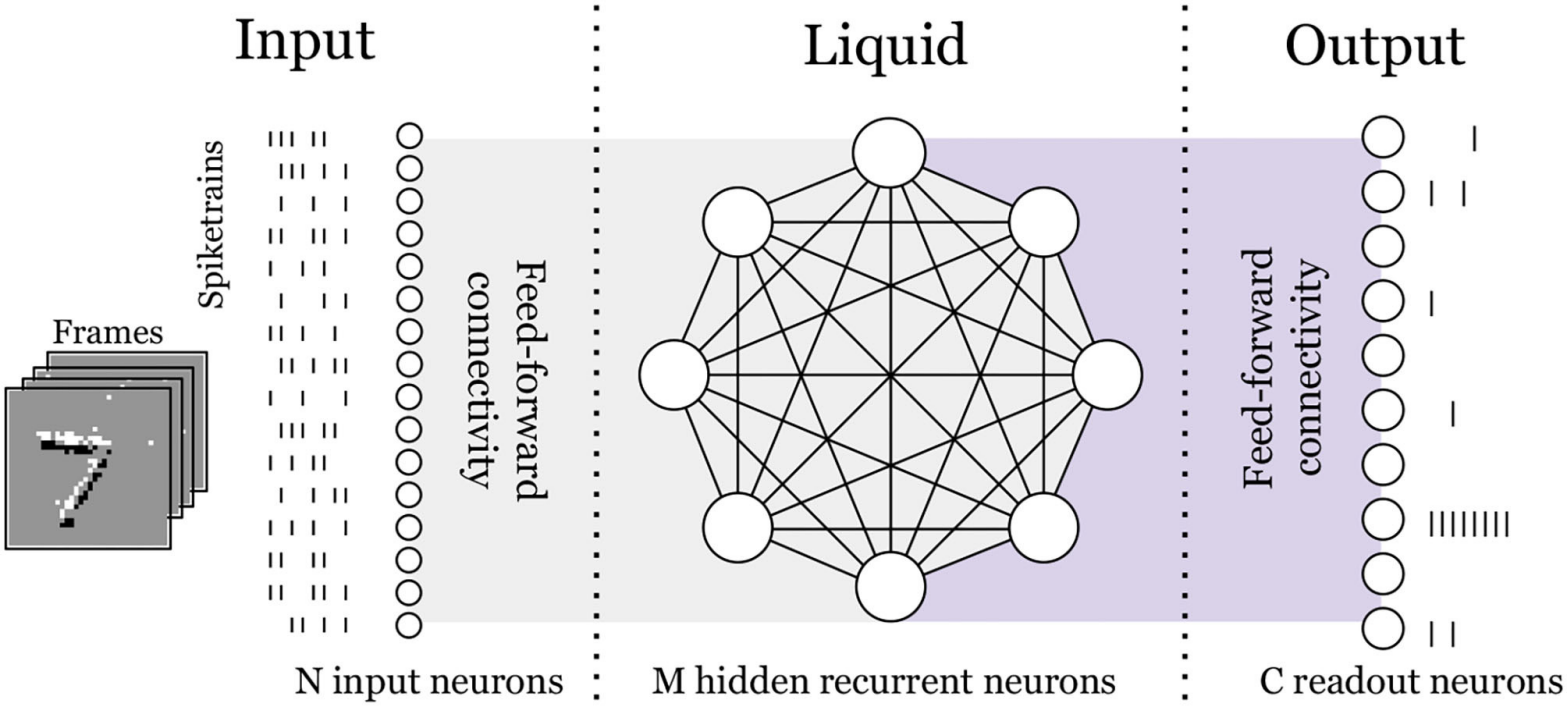
Overview of the LSM architecture for a 2-dimensional temporal input (visualized here as frames) mapped to a 1-dimensional spiketrain. Values of N input neurons and M hidden neurons are smaller than those used in this work, for simplification. Gray shaded area indicates non-trainable synaptic weights and purple shaded area indicates trainable weights.

The spiking neural network that we propose consists of an input layer, a recurrent hidden layer, and a readout layer, also known as Elman RNN (Elman, 1990). The input is a spiking stream with N channels, fully connected to a hidden layer of M neurons, with all-to-all recurrent connections among them (refer to Figure 1). For each time-step *k*, the input current of a neuron in the hidden layer is computed as the weighted summation of the N input spikes plus the weighted summation of the spikes coming from the neighboring neurons in the hidden layer as follows:


(3) $$\begin{array}{l} I\left[k\right]=\overset{N}{\underset{i=1}{\sum }}w_{i}\theta_{i}\left[k\right]+\overset{M}{\underset{j=1}{\sum }}w_{j}\theta_{j}\left[k\right] \end{array}$$

In this equation, *w*_*i*_, *w*_*j*_ are the synaptic strengths from the *i*-th input channel and *j*-th lateral neuron, respectively. Each synapse can be either excitatory (if the weight is positive) or inhibitory (if the weight is negative). The weights are arranged in two connectivity matrices for the input-to-hidden connections *W*_*ih*_ and the hidden-to-hidden connections *W*_*hh*_. The weights are randomly initialized with a uniform distribution from −1/$\sqrt{N}$ to 1/$\sqrt{N}$. θ_*i*_[*k*] and θ_*j*_[*k*] denote the occurrence of a spike on the *i*-th input channel and *j*-th lateral neuron, respectively. The synaptic delay is set equal to the simulation resolution. The spiking mechanism of the *j*-th recurrent neuron is a non-linear function of its membrane potential and is given by:


(4) $$\begin{array}{l} \theta_{j}[k]=\{\begin{array}{ll} 1 & u_{j}[k-1]\geq u_{th}\\ 0 & \text{otherwise} \end{array} \end{array}$$

In order to use the network for a pattern classification task, the hidden layer is fully connected to a readout layer of size C, the number of classes. This readout layer is composed of neurons with the same internal dynamics as the hidden layer but without lateral connectivity. The spike count of the readout is used to compute the cost function of the classification task, as will be addressed in the following section.

### 2.3. Event-Based Dataset

The input of this off-line approach system is the data from a Dynamic Vision Sensor (DVS), which contrary to standard cameras, asynchronously detects brightness changes in the scene. The output of a DVS camera is an event stream, where each event encodes the *x*,*y* location, the time, and the polarity of the brightness change. This representation of the visual information is known as *Address Event Representation* (Sivilotti, 1991; Mahowald, 1992) and provides low power, low latency, and high dynamic range compared to conventional cameras, at the cost of ignoring static information such as shape and color. The sampling rate of this sensor is much higher than that of conventional cameras, about 1MHz, which makes it ideal for real-time dynamic vision applications with low-latency and power system constraints (Linares-Barranco et al., 2019). However, due to the restrictions of the LSM simulation in SpiNNaker, a maximum sampling rate of 1KHz is supported, which is still faster than conventional frame-based cameras.

For validation, in this work, we use the N-MNIST dataset (Orchard et al., 2015), an event-based version of the MNIST handwritten digit dataset (LeCun et al., 1998), with 60K samples, divided into 50K for training and 10K for testing. The N-MNIST was recorded with a DVS mounted in a motorized pan-tilt unit performing a saccade movement. The spatial resolution of the event stream is slightly higher than that of the MNIST dataset, 34 × 34 pixels. Each recording, with an average duration of 300 ms, is converted to 50 frames. For every input, we take both positive and negative changes of illumination as two different channels.

### 2.4. Offline Training With Deep Learning Framework

The sampled event stream is fed to a model of the LSM, built on top of the PyTorch neural network module, where the activation functions and internal computations of the neurons in their forward pass can be easily defined. The engine transforms the customized definition (in this case, a network of neurons whose states are their membrane potential with a common activation function depending on the value of the membrane potential) into a computational graph, tuned for highly efficient parallel computations in the GPU. One additional advantage of PyTorch is that it provides control over which parameters should be trained and which should be kept untrained or “frozen”.

The memory update equation for the PyTorch implementation is based on Equations (2) and (3). Note that the reset mechanism is embedded in this equation as the factor (1−θ_*j*_[*k*]). When a spike occurs, the term multiplied by this factor is reset to zero.


(5) $$\begin{array}{l} u\left[k\right]=u\left[k-1\right]e^{-\frac{1}{\tau_{m}}}\left(1-\theta_{j}\left[k\right]\right)+I\left[k\right] \end{array}$$

We consider a decay term $d=e^{-\frac{\Delta t}{\tau_{m}}}$ with value 0.9. For a Δ*t*= 1.0 ms, this corresponds to a τ_*m*_ of 9.4912 ms.

Using this approach, we were able to train an LSM by connecting the input to a recurrently connected layer of SNNs with Elman Connectivity, whose input-to-hidden and hidden-to-hidden parameters are kept untrained. The bias parameter for the whole network is set to zero. The output layer is a densely connected layer of SNNs with a size equal to the number of classes (10 for N-MNIST). The objective is to maximize the firing rate of the neuron corresponding to the desired output. This is achieved by training the readout layer using the Spatio Temporal Back Propagation (STBP) (Wu et al., 2018) algorithm, a time-dependent generalization of the ANN's backpropagation algorithm. The loss function ℓ across *S* training samples and a time window *T* is defined as:


(6) $$\begin{array}{l} \ell =\frac{1}{S}\overset{S}{\underset{s=1}{\sum }}\|y_{s}-\frac{1}{T}\overset{T}{\underset{t=1}{\sum }}\theta_{s,L}{\|}_{2}^{2} \end{array}$$

where ***y***_***s***_ and **θ_*s,L*_** are the label vector of the *s*-th training sample and its corresponding spike activity vector in the output layer (last layer *L*) after forward propagation, respectively.

Afterward, the trained weights are used for implementation on SpiNNaker.

### 2.5. SpiNNaker Implementation

SpiNNaker is a massively-parallel computer system optimized for the simulation, in real-time, of very large networks of spiking neurons (Plana et al., 2020). Both the system architecture and the design of the SpiNNaker chip were developed by the Advanced Processor Technologies Research Group (APT) at the University of Manchester. Each SpiNNaker chip consists of 18 fully programmable ARM cores.

**Figure 2:**
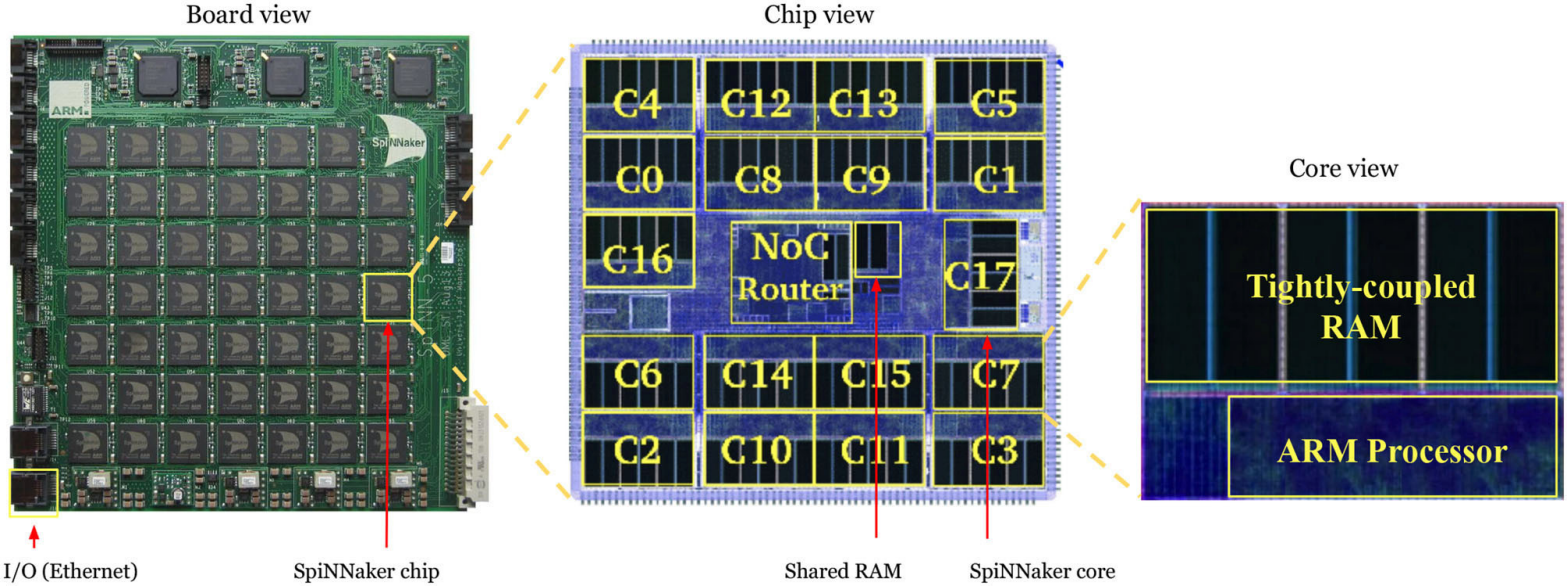
Left: 48-chip SpiNNaker 103 board. Center: View of a single chip, consisting of 18 cores, a shared RAM and a Network-On-Chip router. Right: View of a single core, consisting of a tightly-coupled memory and an ARM processor.

In this work, a SpiNNaker 103 machine (Figure 2) was used. This board comprises 48 SpiNNaker chips, totaling 864 ARM processor cores deployed as 48 monitor processors, 768 application cores, and 48 spare cores. Each application core has two types of RAM: a 32 kB ITCM (instruction tightly coupled memory) for storing instructions and a 64 kB DTCM (data tightly coupled memory) for storing neuron states and parameters. Additionally, each SpiNNaker chip contains a 128 MB SDRAM shared by the 18 cores for storing the synaptic weights. The communication between cores is done through a multicast packet-routing mechanism that mimics the high connectivity found in biological brains. A 100 Mbps Ethernet connection is used for controlling an I/O interface between the computer and the SpiNNaker board. The neurons and synapses are modeled with sPyNNaker (Rhodes et al., 2018), a software package for simulating PyNN-defined spiking neural networks on the SpiNNaker platform.

**Figure 3:**
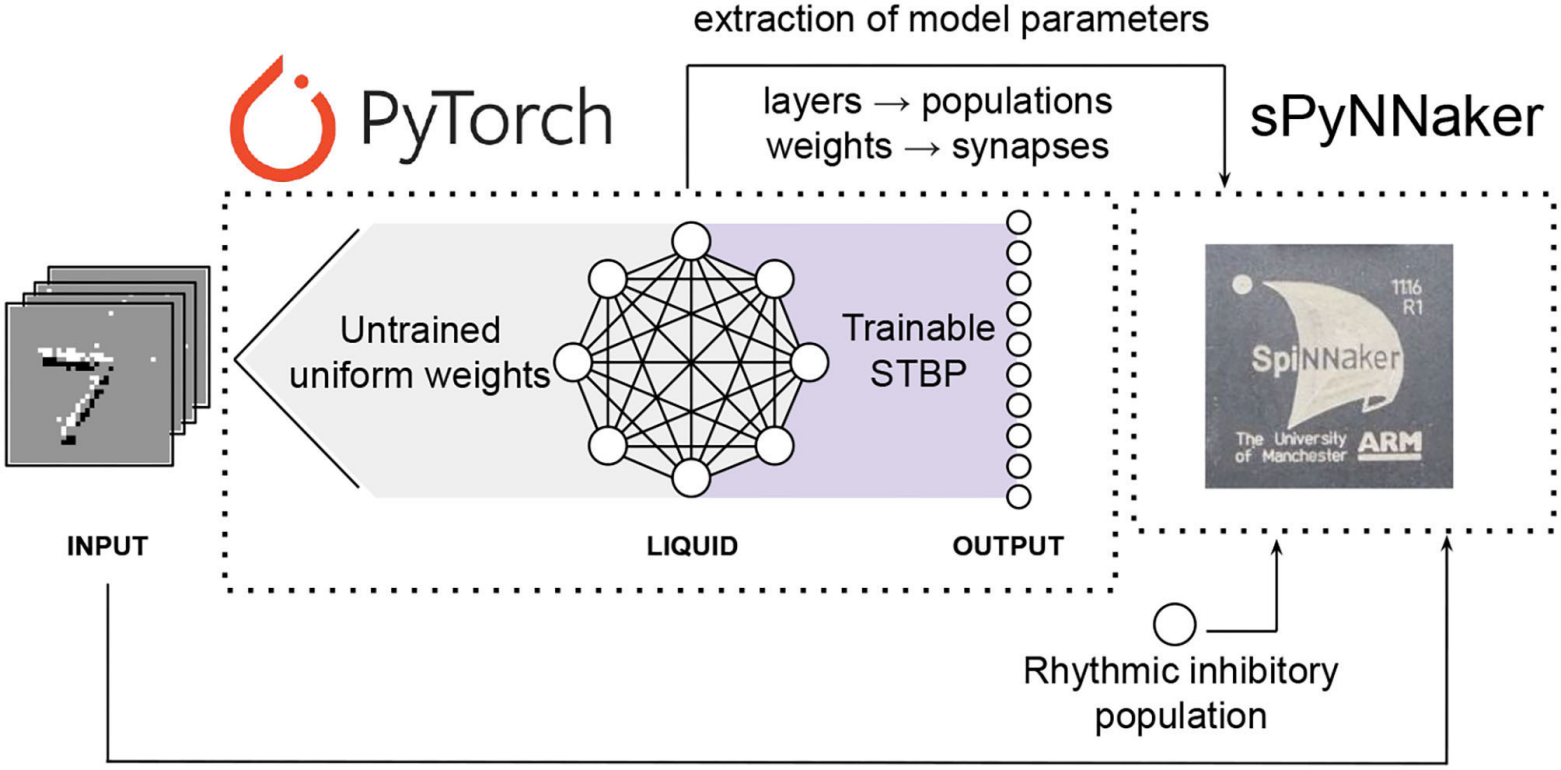
Scheme of the different stages of the neuromorphic LSM classification system proposed in this work. The LSM model is defined in PyTorch, and the output layer is trained using STBP. The trained model parameters are used on an equivalent LSM model defined in sPyNNaker, a PyNN-based API for the SpiNNaker neuromorphic platform. A rhythmic inhibitory population is added to the sPyNNaker model to avoid undesired mixing of responses caused by reverberating activity while performing inference in real time.

**Figure 4:**
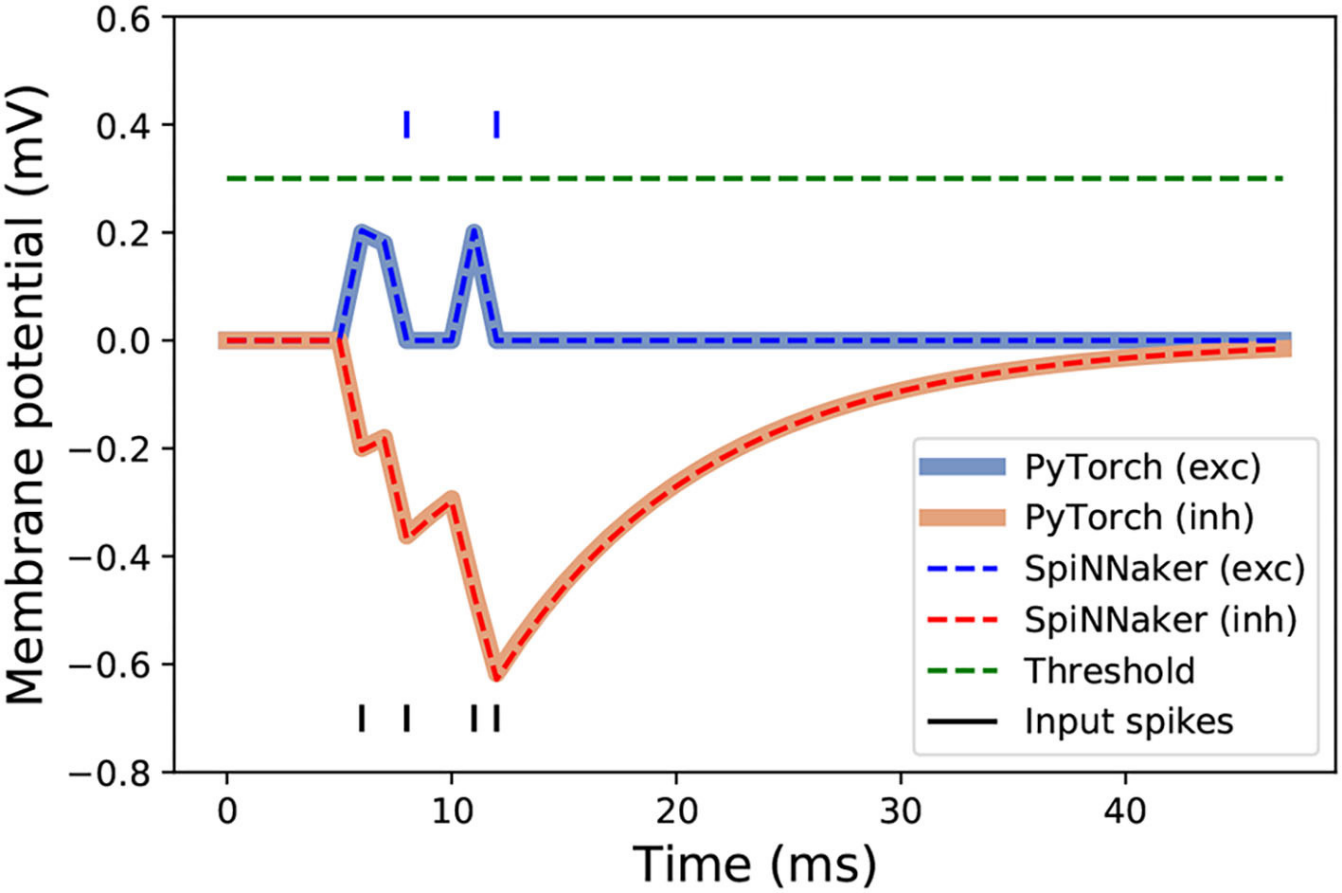
Neuron responses to a single spike train, represented with black vertical segments at the bottom of the figure, in SpiNNaker and PyTorch. Responses of excitatory and inhibitory connections with weights of 0.2 are represented in blue and orange, respectively. The blue line segments above the threshold indicate the occurrence of output spikes from the excited neurons.

A scheme of the different stages of the neuromorphic LSM classification system proposed in this work is shown in Figure 3. After training the LSM model with PyTorch, the platform allows the extraction of the final weights. These are used for reproducing the results from PyTorch with the PyNN-defined SNN and the subsequent implementation on SpiNNaker, provided the neuron and synapse dynamics defined in sPyNNaker match those of PyTorch. This way, the extracted weights are used as synaptic weights in sPyNNaker with exact same values, and contrary to methods that rely on weight conversion (Rueckauer et al., 2017) or train-and-constrain methods (Shrestha et al., 2017), no additional weight preprocessing or approximation is required. The neuron and simulation parameters for the SpiNNaker are given in Table 1. A comparison for single neuron dynamics in both, PyTorch and SpiNNaker, is given in Figure 4. Note that the behavior at the individual neuron level is almost exactly the same.

**Table 1:** SpiNNaker simulation parameters for the proposed LSM.

**Parameter**	**Description**	**Value**
***u*_*reset*_** (mV)	Reset potential	0.0
***u*_*rest*_** (mV)	Resting potential	0.0
***u*_*th*_** (mV)	Threshold	0.3
**τ_*m*_** (ms)	Membrane's time constant	9.4912
***c*_*m*_** (nF)	Membrane's capacitance	0.001
***e*_*rev*_** (mV)	Reversal potential	10,000
***I*_*bias*_** (mA)	Offset current	0.0
**Δ_*t*_** (ms)	Simulation resolution	1.0

To perform inference on SpiNNaker for the whole test set (10K samples), we loaded *via* Ethernet the network parameters and the inputs. The whole network parameters were mapped into the hardware through the high-level sPyNNaker toolbox and each sample was fed to the input population sequentially, with a relaxation of 25 ms after the onset of every sample, where an activity inhibition mechanism (to be explained below) takes place. The duration of the relaxation period is equal to that of the presence of the stimulus and was chosen empirically, as a duration short enough that allowed the inhibition mechanism to work properly.

As the spiking neural network preserves the effect of past inputs in the membrane potential, there should be a way to restore those potentials to their resting values, so the past stimuli do not affect the future stimuli response. Usually, for feed-forward networks, the solution is to let the network's membrane potential decay to the resting potential (as given by the membrane time's constant). However, in recurrent spiking neural networks, as the firing activity persists even after the stimulus is removed due to the recurrent connectivity (see the top of Figure 6), it is necessary to implement an activity reset mechanism. In SpiNNaker, this mechanism exists *via* the high-level Python API SpyNNaker, but it is costly to implement, as it resets not only the membrane's potential but the whole simulation parameters, including the connectivity.

**Figure 5:**
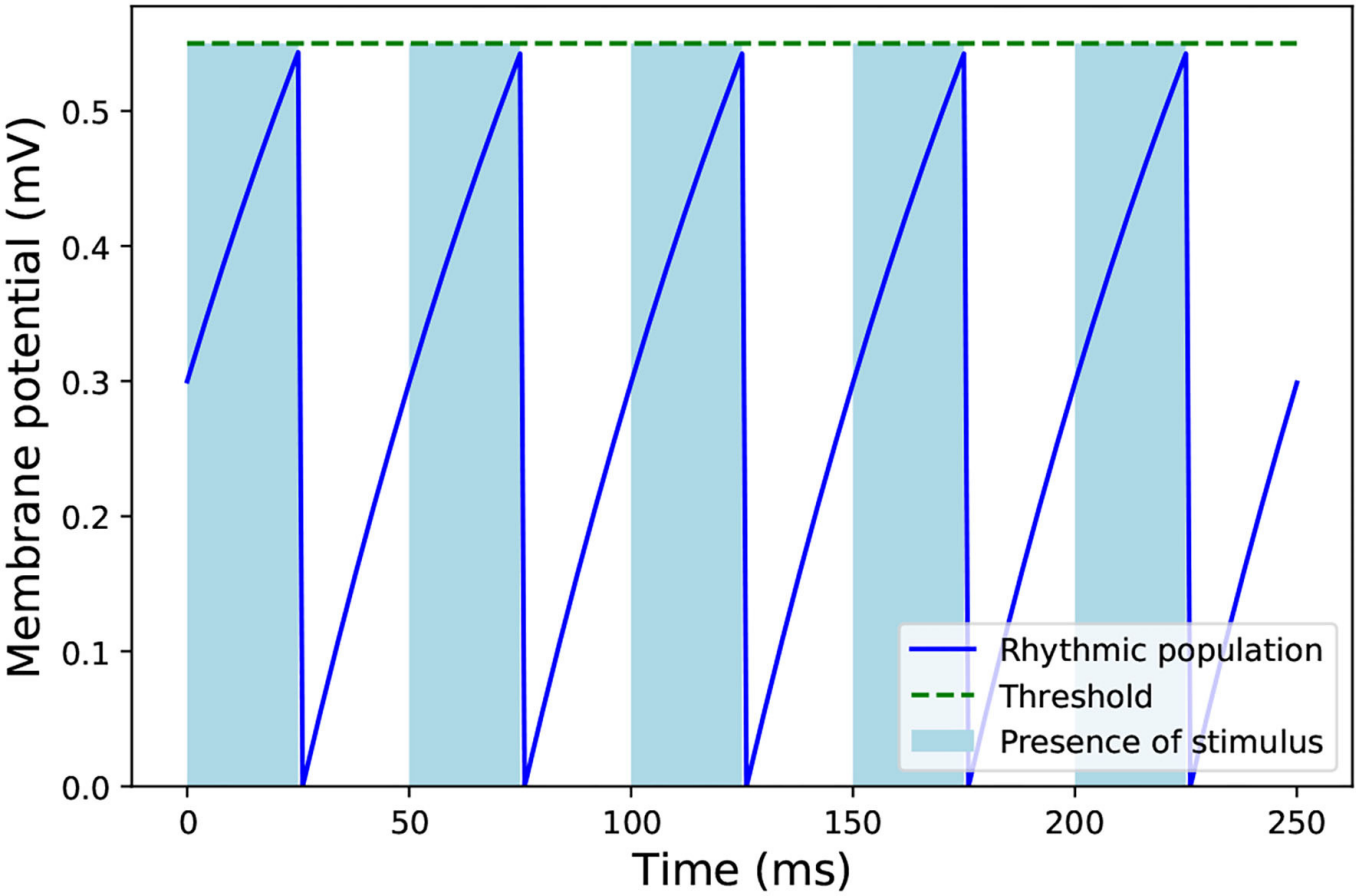
Dynamics of the rhythmic inhibitory neuron connected to the liquid population in SpiNNaker. The light blue stripes indicate the presence of an input stimulus to the network. After the stimulus ends, the inhibitory neuron emits a spike which reduces the activity of the liquid population of the LSM, so it does not affect the next stimulus.

**Figure 6:**
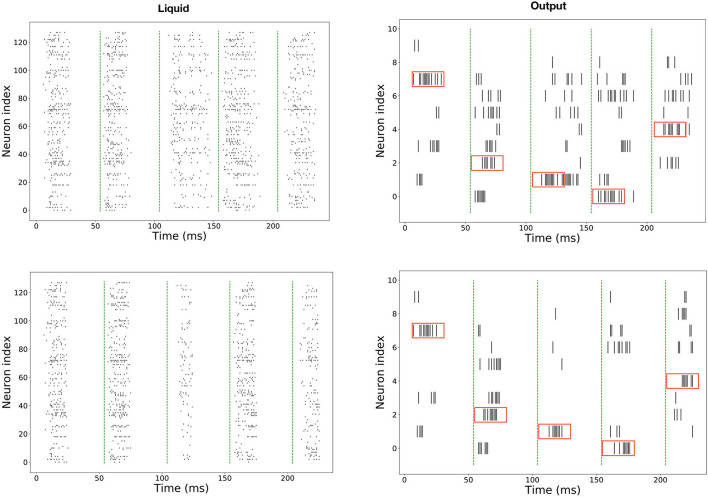
Spiking response in SpiNNaker for five sequential inputs, corresponding to the digits 7, 3, 2, 1, 4. Top: Without a rhythmic inhibitory population. Bottom: With a rhythmic inhibitory population attached to the liquid population. The red boxes in the readout indicate the expected response, and the green dashed lines mark the start of new inputs.

Given the above, an inhibitory population of 1 neuron was added to the network, with one-to-all connectivity to the hidden layer. This neuron fires at regular intervals to coincide with the final time step of each sample as shown in Figure 5. The bottom half of Figure 6 shows the effect of this homeostatic population on the overall behavior of the network. This inhibitory neuron is a technical solution to the implementation of recurrent spiking neural networks in SpiNNaker, so the partitioning and mapping occur only once and the inference process is smooth. It does not have any measurable effect on the accuracy of the LSM, as long as the timing of the inhibitory neuron coincides with the end of each stimulus.

### 2.6. Weight Quantization

**Figure 7:**
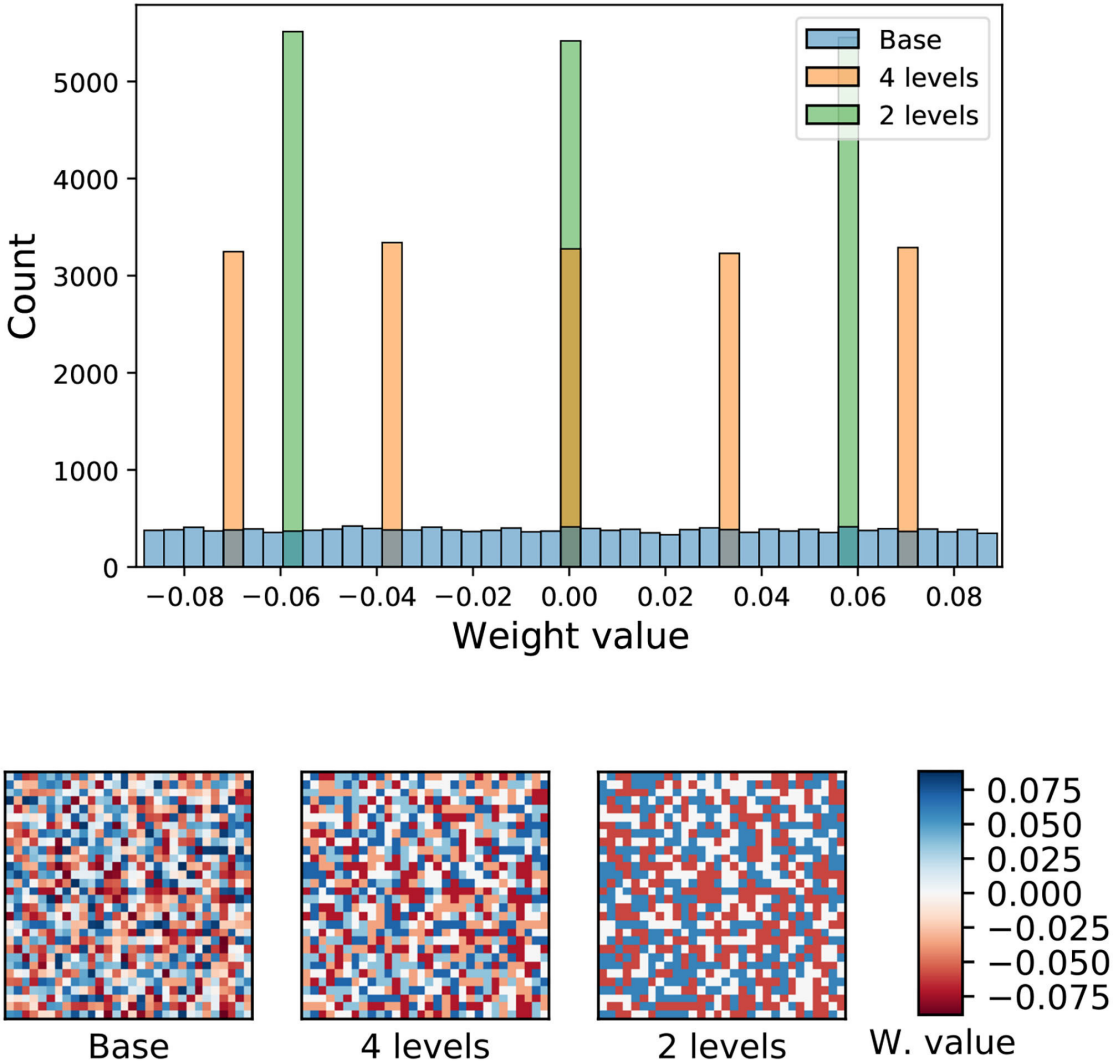
Top: Weight distribution of the liquid-to-liquid synapses for three different precisions. Bottom: 2D representation of the weight values for the three quantization schemes. For this example, a liquid population of 128 was considered. Each resulting 128 × 128 image was cropped for better visualization.

Weight quantization is a method to reduce the memory requirements of ANNs in order to enable faster and more efficient inference in hardware without significantly compromising accuracy (Han et al., 2015). This method can also be useful for neuromorphic computing as some of the available hardware platforms, such as Neurogrid (Benjamin et al., 2014), BrainScales (Schemmel et al., 2010), and TrueNorth (Merolla et al., 2014) operate with reduced precision in their synaptic weights: 13-, 4-, and 1-bit, respectively. Although SpiNNaker supports up to 32-bit fixed-point precision, we were interested in the behavior of our proposed LSM with a more reduced weight precision, considering the small size of the memory where synaptic weights per chip are stored, 128 MB. To this end, the input-to-hidden and hidden-to-hidden weights were quantized following the rule:


(7) $$\begin{array}{l} w_{q}=s\cdot round\left(\frac{w\cdot 2^{b}}{s}\right) \end{array}$$

where *w*_*q*_ is the quantized weight, *w* is the original weight, *b* is the number of bits of the resulting weight distribution and *s* is a normalization scale obtained from the original weight distribution. Figure 7 shows the weight distribution of the liquid-to-liquid synapses when quantization is applied for a liquid population of 128 neurons.

The weight quantization is training-agnostic, because the two sets of internal weights which are quantized, input-to-hidden and hidden-to-hidden, are not involved in the training process (as imposed by the Liquid State Machine model). The only set of weights that is trained is the hidden-to-output, but these weights were not quantized. In our experiments, we quantized the internal weights before and after training (in PyTorch) yielding no significant differences in the final classification accuracy.

## 3. Results

We have trained LSM of sizes ranging from 128 to 4,096 neurons and implemented them in SpiNNaker. For measuring the performance of the implementation, the whole test set of the N-MNIST (10,000 samples) was propagated in both PyTorch and SpiNNaker. An example of the spiking activity in the hidden and readout layers is shown in Figure 6. These are raster plots displaying the spike times of each neuron for the first ten examples. An image is considered to have been classified correctly if the neuron of the readout layer associated with its label displays a higher firing rate than the other neurons while the stimulus is on. The total simulation time for each input is 50 ms. A summary of the results for different sizes of the hidden layer is given in Table 2. The only work found in the literature that uses LSM for this dataset is Tian et al. (2021). In their work, they report a 90.1% accuracy for a single liquid of size 1,000 and a maximum accuracy of 92.5% for an ensemble of liquids using neural architecture search. Our work reaches a top accuracy of 94.43% for a liquid of size 4,096. Additionally, this is the first reported use of LSM for a complex spatio-temporal task in a neuromorphic platform. The best accuracy for SpiNNaker is 93.9%. It can be seen that there is a small variability in the performance of both platforms, due to small differences in the precision of the models and the inherent noise of the SpiNNaker implementations, possibly caused by dropped packets due to congestion in its interconnect (Plana et al., 2020).

**Table 2:** Results for different sizes of the hidden layer.

**Size**	**PyTorch**	**SpiNNaker**
128	74.7	75.24
256	82.79	82.26
512	87.96	88.12
1024	90.19	90.29
2048	92.88	92.36
4096	94.43	93.9

**Figure 8:**
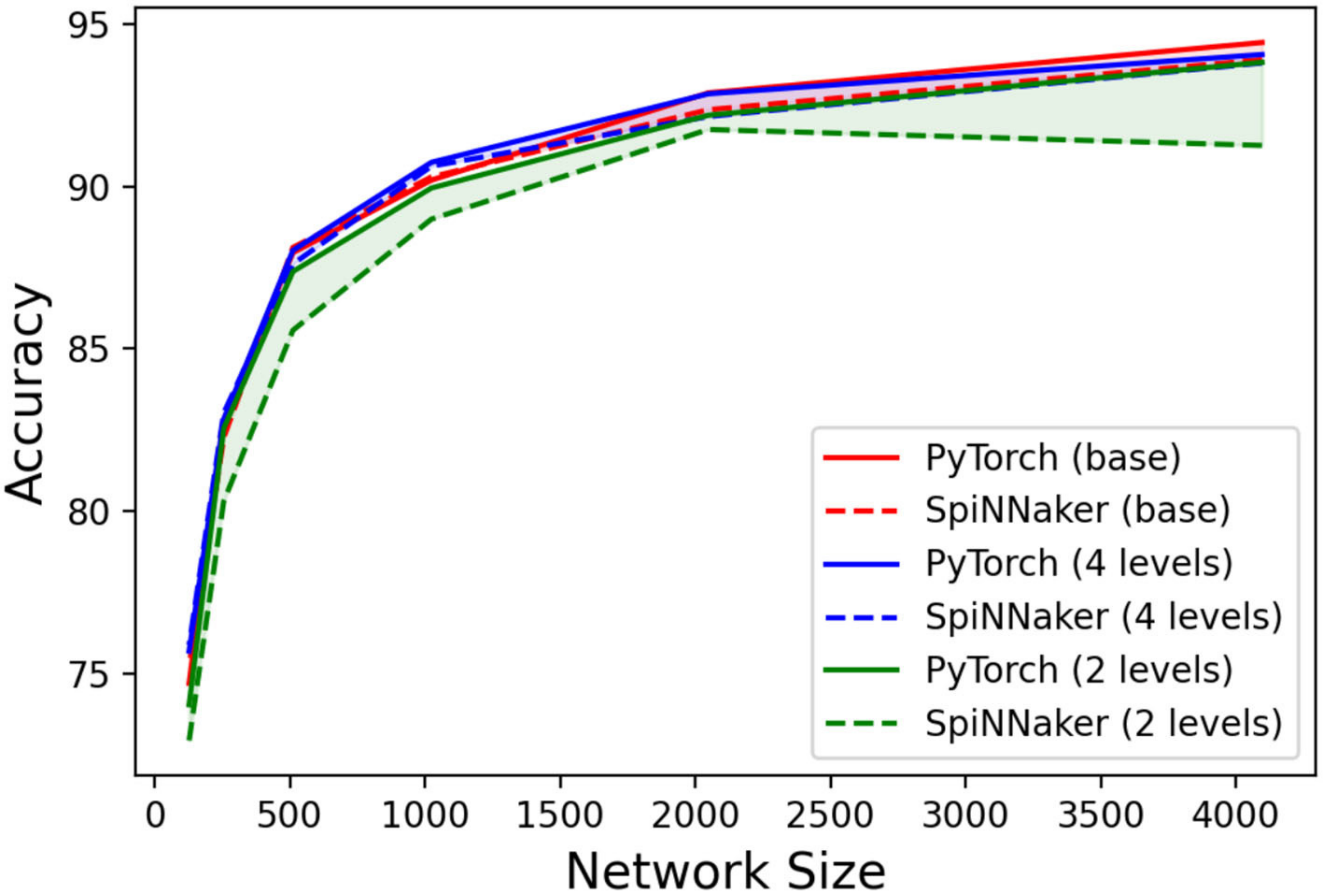
Accuracies per network size (number of neurons in the liquid population) for three different weight precisions implemented in PyTorch and SpiNNaker.

Additionally, we performed tests with quantized weights, whose results are summarized in Figure 8. It can be observed that the use of weight quantization does not have a significant impact on the accuracy of the LSM in PyTorch. Regarding the SpiNNaker implementation, only the two-level quantization (or weight binarization) has a noticeable impact on the performance, especially with higher network sizes.

## 4. Discussion and Future Outlook

In this article, we introduced a method to facilitate the inference of Recurrent Spiking Neural Networks on the SpiNNaker neuromorphic platform. This was validated by implementing a Liquid State Machine for an event-driven classification task, the N-MNIST, achieving the best-known accuracy results for such architecture and dataset. Additionally, we showed that the accuracy is not significantly affected when the simulated weights are constrained by quantization.

Regarding the speed of this neuromorphic implementation, the wall-clock time required for a single inference depends on the *time scale factor* parameter used in SpiNNaker, which allows slowing down the simulations for a more reliable operation. Our simulations are designed so every inference takes 50 ms (25 ms for classifying the input and 25 ms for the inhibition period in preparation for the next input). However, in our best-reported results, a time scale factor of 5 was used, meaning a wall-clock time inference of 250 ms. This is far from ideal, and we encourage the community to find ways to implement recurrent connectivity in neuromorphic hardware which is robust to packet loss, so the inference can approach real-time.

Considering the size of the networks implemented in this work, it is small for the potential of the SpiNNaker, which can simulate 255 LIF neurons per core, approximately 195K neurons in the 48-chip board. However, in our experiments we observe that the accuracy starts dropping beyond 10 neurons per core, limiting the maximum network sizes below 7,680 neurons. We hope that future works aiming for fast, accurate inference of large spiking neural networks in this platform build upon our work.

Additionally, in future works, we would like to adapt our methodology to perform on-chip training and validate it in datasets with richer dynamical content. We will favor the use of biologically-feasible time-coded instead of rate-coded learning rules as it would reduce the spiking activity in the readout layer. We believe this work can be found valuable in the quest for building and implementing highly performing Spiking RNNs in neuromorphic processors which in turn would be a seed for future developments of energy-efficient multi-scale processing applications.

## Data Availability Statement

Publicly available datasets were analyzed in this study. This data can be found here: https://www.garrickorchard.com/datasets/n-mnist.

## Author Contributions

AP-S, HR-G, TS-G, and BL-B conceived and planned the experiments, contributed to the interpretation of the results, and contributed to document preparation. AP-S and HR-G carried out the experiments. AP-S, TS-G, and BL-B planned and carried out the simulations. AP-S took the lead in writing the manuscript. All authors provided critical feedback and helped shape the research, analysis, and manuscript. All authors contributed to the article and approved the submitted version.

## Funding

This work was supported by EU H2020 grant 871371 (MeM-Scales), by Spanish grant from the Ministry of Science and Innovation PID2019-105556GB-C31 (NANOMIND) with support from the European Regional Development Fund), and by CONACYT scholarship number 688116/578600.

## Conflict of Interest

The authors declare that the research was conducted in the absence of any commercial or financial relationships that could be construed as a potential conflict of interest.

## Publisher's Note

All claims expressed in this article are solely those of the authors and do not necessarily represent those of their affiliated organizations, or those of the publisher, the editors and the reviewers. Any product that may be evaluated in this article, or claim that may be made by its manufacturer, is not guaranteed or endorsed by the publisher.
